# Difluprednate *versus* prednisolone acetate for inflammation following cataract surgery in pediatric patients: a randomized safety and efficacy study

**DOI:** 10.1038/eye.2016.132

**Published:** 2016-07-01

**Authors:** M E Wilson, H O'Halloran, D VanderVeen, J Roarty, D A Plager, K Markwardt, K Gedif, S R Lambert

**Affiliations:** 1 Storm Eye Institute, Medical University of South Carolina, Charleston, SC, USA; 2 Rady Children's Hospital, San Diego, CA, USA; 3 Boston Children's Hospital, Harvard Medical School, Boston, MA, USA; 4 Department of Ophthalmology, Children's Hospital of Michigan, Wayne State University School of Medicine, Detroit, MI, USA; 5 Indiana University Medical Center, Indianapolis, IN, USA; 6 Alcon Research Ltd, Fort Worth, TX, USA; 7 Department of Ophthalmology, Emory University School of Medicine, Atlanta, GA, USA

## Abstract

**Purpose:**

To evaluate safety and efficacy of difluprednate 0.05% ophthalmic emulsion for treatment of postoperative inflammation after cataract surgery in pediatric patients.

**Methods:**

This was a phase 3B, multicentre, randomized, double-masked, active-controlled study of patients aged 0–3 years who underwent uncomplicated cataract surgery in one eye, with/without intraocular lens implantation. Patients were randomized to receive difluprednate 0.05% four times daily or prednisolone acetate 1% for 14 days post surgery, followed by tapering for 14 days. Safety included evaluation of adverse events. Primary efficacy was the proportion of patients with an anterior cell grade of 0 (no cells) at day 14; secondary efficacy was a global inflammation score.

**Results:**

Forty patients were randomized to each treatment group. Adverse drug reactions included corneal oedema (difluprednate 0.5%, *n*=1; prednisolone acetate 1%, *n*=0) and increased intraocular pressure or ocular hypertension (*n*=2/group). Mean intraocular pressure values during treatment were 2–3 mm Hg higher with difluprednate 0.05% compared with prednisolone acetate 1% mean values were similar between groups by the first week after treatment cessation. At 2 weeks post surgery, the incidence of complete clearing of anterior chamber cells was similar between groups (difluprednate 0.05%, *n*=30 (78.9%); prednisolone acetate 1%, *n*=31 (77.5%). Compared with prednisolone acetate 1%, approximately twice as many difluprednate 0.05%-treated patients had a global inflammation assessment score indicating no inflammation on day 1 (*n*=12 (30.8%) *vs n*=7 (17.5%) and day 8 (*n*=18 (48.7%) *vs n*=10 (25.0%).

**Conclusions:**

Difluprednate 0.05% four times daily showed safety and efficacy profiles similar to prednisolone acetate 1% four times daily in children 0–3 years undergoing cataract surgery.

## Introduction

In children with congenital cataract who are otherwise healthy, early cataract surgery can result in good corrected visual acuity and binocular function.^1, 2^ However, compared with older children and adults, young children tend to have a more substantial inflammatory response to cataract surgery.^3, 4^ This inflammatory response, which is characterized by the presence of cells and flare in the anterior chamber, occurs when the blood–aqueous barrier is compromised.^5^ If left untreated, inflammation can interfere with the child's visual rehabilitation and may lead to further complications.^6, 7, 8^ Treatment with anti-inflammatory agents during the postoperative period allows for more rapid resolution of inflammatory symptoms and improved patient comfort.^9, 10^

Controlled studies have demonstrated that topical steroids are effective and have a favourable safety profile in suppressing ocular inflammation and reducing the long-term likelihood of vision impairment when administered at the time of surgery and during the weeks afterwards.^10, 11^

Difluprednate ophthalmic emulsion 0.05% is a topical ocular corticosteroid that has been approved since 2008 for use in adults, initially for treatment of inflammation and pain associated with ocular surgery, and subsequently for treatment of endogenous anterior uveitis.^12, 13^ Recent studies in adults have shown that difluprednate treatment is associated with rapid resolution of both inflammation and pain associated with ocular surgery.^9^ Phase 3 studies in adults have shown that difluprednate 0.05% four times daily is well tolerated and noninferior to prednisolone acetate 1% eight times daily for treatment of endogenous anterior uveitis.^12, 14^

Children have a greater propensity for postoperative inflammation than adults.^3, 8, 15^ The safety of topical difluprednate after ocular surgery in children has not been previously established.^16^ The current study was designed in response to a written request by the US Food and Drug Administration in February 2009 to evaluate the safety of difluprednate 0.05% in children aged 0–3 years for treatment of postoperative inflammation following cataract surgery. In our study, prednisolone acetate 1% was selected for comparison because it is currently widely accepted in the United States as standard care in the treatment of inflammation following ocular surgery in children and adults.

## Patients and methods

### Study design

This was a phase 3B, multicentre, randomized, double-masked, parallel-group, active-controlled study (NCT01124045) initiated in August 2010 and completed in April 2012. It was designed to compare the safety and efficacy of difluprednate 0.05% (Durezol ophthalmic emulsion, Alcon Laboratories, Inc., Fort Worth, TX, USA) and prednisolone acetate 1% (Pred Forte ophthalmic suspension, Allergan, Inc., Irvine, CA, USA) in the treatment of inflammation following cataract surgery in children aged 0–3 years.

All investigative sites were located in the United States. Institutional Review Board/Ethics Committee approval was obtained before the start of the study. The study was performed in compliance with the ethical principles of the Declaration of Helsinki and Good Clinical Practice, and informed consent was obtained from a parent or legal guardian before enrolment of each patient.

Patients were randomly assigned to treatment groups in accordance to a planned ratio of 1 : 1. Randomization numbers were generated using computer software (PROC PLAN, SAS Institute, Inc., Cary, NC, USA). The protocol was amended to include stratification by race (white and non-white) in randomization procedures to ensure a balance across treatment group. Patients, caregivers, and investigators were masked to the medication being instilled. Because prednisolone acetate 1% is a suspension that needs to be shaken before instillation, parents or legal guardians of patients were instructed to shake the assigned medication bottle before instillation to preserve masking.

### Patients

Children (age 0–3 years) enrolled in the study were scheduled to undergo uncomplicated cataract surgery in one eye, with or without intraocular lens implantation. Use of contact lenses for postsurgery refractive correction when operating on young infants with a unilateral cataract who are <7 months of age was recommended by the authors of a prospective study published^1^ while the study was ongoing. Hence, an amendment was made to the original protocol to allow the inclusion of children wearing contact lenses to correct aphakia post surgery. Exclusion criteria included: active uveitis, neoplasia, or any active or suspected viral, bacterial, or fungal disease in the study eye; use of any topical medication in the study eye within 7 days before surgery (except those required for ocular examination or preoperative preparation); systemic use of steroids or non-steroidal anti-inflammatory drugs; a history of steroid-induced increases in intraocular pressure (IOP); current use of medication for ocular hypertension or glaucoma in the study eye; post-traumatic cataract; suspected permanent low vision or blindness in the non-study eye; human immunodeficiency virus, acquired immunodeficiency syndrome, or diabetes.

### Interventions

All patients completed a screening visit within the 14 days preceding or on the day of surgery (day 0). Study treatment began on day 0 with one drop of the randomized medication instilled into the operated eye immediately following surgery. Following surgery, the parent or legal guardian of each patient was instructed to instil one drop of the randomized study medication into the affected eye four times daily, beginning on day 1, for 14 days after surgery, followed by tapering for 14 days. The treatment regimen during tapering depended on the investigator's assessment of the response to treatment. The treatment regimen used in the present study in both treatment arms (one drop four times daily) was based upon current dosing recommendations for patients following cataract surgery.^13^

### Study visits

Patients were evaluated for safety and efficacy on days 0, 1, 8 (±1 day), and 15 (±2 days), and at the end of study drug treatment (day 29 ±2 days). Additional safety visits occurred 1 week (+2 days) and 3 months (+1 week) after the last dose of study drug, the last visit occurring at the earliest on day 92, and at the latest on day 99.

### Safety evaluations

Safety was evaluated throughout the study by recording adverse events (AEs) as coded according to the Medical Dictionary for Regulatory Activities. Recorded AE data included incidence, seriousness, relationship to treatment, association with study discontinuation, and individual characteristics (eg, severity, onset, duration). Mean and change from baseline in visual acuity and IOP were also evaluated. Change from baseline in fundoscopic parameters (vitreous (⩾1 unit increase in grade from baseline), retina/macula/choroid (an increase to a score of grade 2, if not present at baseline), and optic nerve (⩾1 unit increase in grade from baseline), evidence of postoperative bacterial or fungal infection, and change from baseline (⩾1 unit increase) in ocular sign parameters (lids, cornea, iris, sclera, conjunctiva, lid margins, and lens) were also recorded. Ocular signs were included in the safety analysis.

### Efficacy evaluations

The primary efficacy end point was the number and percentage of patients with an anterior cell grade of 0 (ie, no cells) at the end of the 14-day treatment period (day 15±2 days). Secondary efficacy end points were assessed at all study visits and included a 3-point global assessment of inflammation scores (0 (clear), 1 (improving satisfactorily), and 2 (not improving or worsening, or withdrawal from study indicated to allow institution of appropriate alternative therapy), and the individual components that informed the global assessment score. The individual components comprised: anterior chamber cell grade, anterior chamber flare grade, corneal clarity, wound integrity, conjunctival injection, ciliary/limbal injection, chemosis, hypopyon, vitritis, lens reproliferation (described), and symptoms (photophobia and lacrimation). With the exception of anterior chamber cell grade, which was reported using a 5-point scale ranging from 0 (0 cells) to 4 (⩾50 cells), the individual components were reported using a 4-point scale: 0, absent; 1, mild; 2, moderate; and 3, severe.

### Statistical analyses

To allow for at least a 95% chance of detecting an AE with a 10% incidence rate, a minimum sample size of 30 patients per group (difluprednate 0.05% or prednisolone acetate 1%) was required. No inferential statistical analysis was planned, and data were summarized using descriptive statistics only. Assessment of efficacy was based primarily on the number and percentage of patients in each treatment group with an anterior chamber cell grade of 0 at day 15 and, secondarily, on the global assessment score of postoperative inflammation and the individual components of the global assessment score (number and percentage of patients in each treatment group in each score category at each visit). All statistical analyses were performed using Statistical Analysis System (SAS) software. The intent-to-treat data set included all patients who received study medication, and had at least one scheduled on-therapy study visit. Primary efficacy assessments were based upon the intent-to-treat data set.

## Results

### Patient disposition and demographics

A total of 80 children were enrolled (difluprednate 0.05%, *n*=40; prednisolone acetate 1%, *n*=40; Supplementary Figure 1). One child randomized to difluprednate 0.05% had consent withdrawn before receiving study medication and was excluded from the intent-to-treat (*n*=79), and safety (*n*=79) populations. Eligible children's ages ranged from 10 days to a maximum of 47 months, although the majority of children were aged between 28 days and 23 months in all analysis data sets. Children in the category of 28 days to 23 months included all children of at least 28 days of age who had not reached their second birthday. Those in the 2–3-year category included all children who had reached their second birthdays even if they had reached their third birthday. There were no clinically relevant differences between the patient groups in terms of age, sex, ethnicity, race, or iris colour (Table 1).

### Safety

#### Adverse events

In all, 2 (5.1%) children in the difluprednate 0.05% group and 2 (5.0%) children in the prednisolone acetate 1% group experienced at least one AE related to treatment. Of those, children experienced AEs involving IOP increases (difluprednate 0.05%, *n*=2, 5.1% prednisolone acetate 1%, *n*=1, 2.5%), and 1 child (prednisolone acetate 1%, *n*=1, 2.5%) experienced a Medical Dictionary for Regulatory Activities–coded AE of ocular hypertension (defined as IOP >21 mm Hg). An additional AE of increased IOP occurred in the difluprednate 0.05% group, but was reported by the investigator as unrelated to treatment. The third treatment-related AE report was of corneal oedema (Table 2) that occurred in the same patient from the difluprednate 0.05% group experiencing an AE of increased IOP.

Nonfatal serious adverse events (SAEs) were reported for 8 (20.5%) children in the difluprednate 0.05% group, and 11 (27.5%) children in the prednisolone acetate 1% group. The SAEs included ‘medical observation' (difluprednate 0.05%, *n*=6, 15.4% prednisolone acetate 1%, *n*=11, 27.5%), cortical cataract that was resolved by vitrectomy surgery (difluprednate 0.05%, *n*=1, 2.6% prednisolone acetate 1%, *n*=0), a metabolic and nutrition disorder that was Medical Dictionary for Regulatory Activities–coded as ‘failure to thrive' (difluprednate 0.05%, *n*=0; prednisolone acetate 1%, *n*=1, 2.5%), and increased IOP (difluprednate 0.05%, *n*=1, 2.6% prednisolone acetate 1%, *n*=0). The high incidence of ‘medical observation' as an SAE in this study occurred because overnight hospitalization is the standard of care for neonatal patients after surgery/anesthesia, and hospitalization automatically triggers recording of an SAE. None of the SAEs resulted in discontinuation of treatment.

#### Intraocular pressure

**Figure 1:**
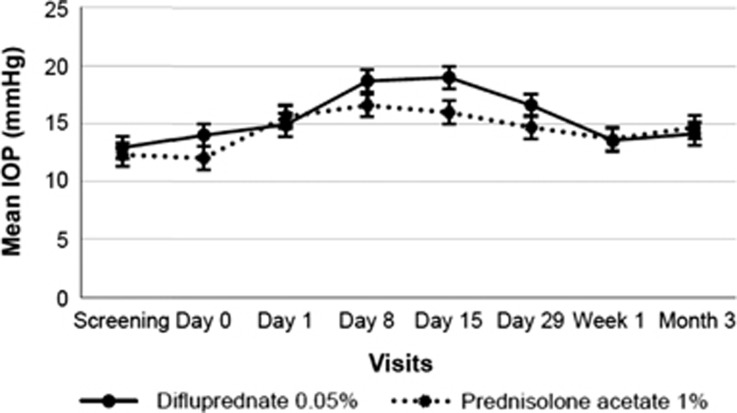
Change in mean IOP (mm Hg) following difluprednate and prednisolone treatment after cataract surgery. IOP, intraocular pressure.

On days 8, 15, and 29, mean IOP values were 2–3 mm Hg higher in children treated with difluprednate 0.05% than in those who received prednisolone acetate 1%, but mean values in the two groups were similar by the first week after treatment cessation and remained similar at 3 months after treatment (Figure 1). Two children (one from each treatment group) reported an IOP ⩾40 mm Hg at unscheduled visits between study days 15 and 29 (child in difluprednate 0.05% group, IOP=41 mm Hg) and between study days 1 and 8 (child in prednisolone acetate 1% group, IOP=43 mm Hg).

#### Other safety variables

There were no clinical differences in visual acuity data between treatment groups. A higher proportion of children receiving prednisolone acetate 1% reported changes from baseline in optic nerve (increase in grade of ⩾1 unit from baseline: difluprednate 0.05%, *n*=0; prednisolone acetate 1%, *n*=1, 3.4%) and retina/macula/choroid (Grade 2; difluprednate 0.05%, *n*=0; prednisolone acetate 1%, *n*=2, 6.7%). Changes in vitreous (increase in grade from baseline of ⩾1 unit) were similar in the difluprednate 0.05% and prednisolone acetate 1% groups (*n*=2, 7.1% and *n*=1, 3.2%, respectively). There were no postoperative bacterial or fungal infections reported. For each ocular sign parameter (lids, lid margins, conjunctiva, cornea, sclera, iris/anterior chamber), more children treated with prednisolone acetate 1% had increases from baseline of ⩾1 unit at any visit than patients treated with difluprednate 0.05%.

### Efficacy

#### Primary efficacy end point

The two groups showed similar results for the primary efficacy end point: complete clearing of anterior chamber cells (anterior cell grade 0) on day 15 was recorded in 30 patients (78.9%) in the difluprednate 0.05% group and in 31 patients (77.5%) in the prednisolone acetate 1% group.

#### Secondary efficacy end points

A higher proportion of difluprednate-treated children had a global inflammation assessment score of 0 (no evidence of postoperative inflammation) on days 1 and 8 when compared with patients treated with prednisolone acetate (Table 3; day 1, 12 (30.8%) *vs* 7 (17.5%) children, respectively; day 8, 19 (48.7%) *vs* 10 (25.0%) children, respectively). The proportions of children who had a global inflammation assessment score of 0 on day 15 were comparable between the two groups (difluprednate 0.05%, *n*=22, 56.4% prednisolone acetate 1%, *n*=20, 50.0%).

On day 15, similar efficacy was reported between the two treatment groups for the individual components that informed the global inflammation assessment, including conjunctival injection, cillary/limbal injection, chemosis, and hypopyon (Table 4). Furthermore, by day 15, a similar proportion of patients in both groups had an anterior chamber flare grade of 0 (difluprednate 0.05%: *n*=29, 74.4% prednisolone acetate 1%: *n*=28 70.0%); 38 patients in both groups (difluprednate 0.05%, 97.4% prednisolone acetate 1%, 95.0%) were completely clear of inflammation, as indicated by absence of photophobia; 39 patients in both groups (difluprednate 0.05%, 100.0% prednisolone acetate 1%, 97.5%) were completely clear of cells in the vitreous; and ⩾90% of patients in both groups had complete corneal clarity (Table 4). As stated in the primary efficacy end point, the percentage of children with an anterior chamber cell grade of 0 was similar in the two groups.

## Discussion

In a population of children (aged 0–3 years) undergoing cataract surgery, the overall incidences of AE of topical difluprednate 0.05% and prednisolone acetate 1% four times daily were similar, with no AEs leading to discontinuation. Serious adverse events were reported in both treatment groups, although no new or unexpected safety concerns were observed. Hospitalization for medical observation, the most commonly reported SAE in the current study, was associated with hospital policies of admission for infants after general anaesthesia. As a result, the number of SAEs recorded in this study may be higher than in an adult patient study^9^ in which postoperative hospital admission would not be automatic. Excluding hospitalization SAEs, and considering expected outcomes based on the characteristics of this population following cataract surgery^4, 17^ and previous experience with topical ocular steroids,^18^ no unexpected safety concerns were detected in our study. In our study, the overall percentages of patients reporting difluprednate-related increased IOP as an AE (5.1%) were similar to those reported by Smith *et al*^19^ (6.2%).

In our study, a substantial elevation in IOP ⩾40 mm Hg was found in two children, one from each treatment group.^20, 21^ Elevated IOP is a known potential adverse reaction to steroid use,^22^ and is listed in the warnings section of the package insert for this drug class. Based upon previous experience with topical ocular steroids, class labelling, and the characteristics of the patient population in the current study, the incidence of increased IOP is not unexpected.

The balance between efficacy and risk of increased IOP is an important challenge in the management of all cataract surgery patients.^23^ In our study, on day 8, when mean IOP values were higher in children treated with difluprednate 0.05% than in those treated with prednisolone acetate 1%, a higher percentage of difluprednate-treated children were completely clear of postoperative inflammation than patients treated with prednisolone acetate (48.7% *vs* 25.0%, respectively). These data suggest that although mean IOP values were initially higher with difluprednate 0.05% treatment, this came with a more rapid control of inflammation than was achieved with prednisolone acetate 1%. This is similar to the findings of Sheppard *et al*,^14^ in an endogenous anterior uveitis study.^14^

When comparing the safety and efficacy of difluprednate 0.05% with prednisolone acetate 1%, differences in drug delivery and formulations should be taken into consideration. Prednisolone acetate 1% is a suspension and must therefore be shaken before application; difluprednate 0.05% is an emulsion that does not require shaking. This difference in formulation may be significant because if the prednisolone acetate 1% suspension does not receive adequate shaking before application, an incorrect dose may be delivered to the eye.^24^ Correct dosage is of particular importance in young children because, compared with adults, complications after cataract surgery have a greater impact on long-term visual outcomes.^8^ Emulsions, such as difluprednate 0.05%, and gel formulations may therefore be preferred to suspensions because of the accuracy that they offer from dose to dose.^24, 25^

Inclusion in the current study of aphakic infants treated with a contact lens is important, as contact lens use is commonly used for correction of aphakia in infants.^1^ In this study, application of the study medication in the affected eye was possible while the contact lens remained in place. Analysis of AEs by age showed no clinically relevant differences among the individual AE characteristics between the overall safety population and patients in each age category for any treatment group. Thus, the inclusion of children (including those <7 months of age) with contact lenses resulted in no new or unexpected safety concerns that would alter the safety profile of difluprednate 0.05% in pediatric patients dosed four times daily for at least 14 days.

This study had a number of important limitations. Children aged 0–3 years are often difficult to examine; nonetheless, the surgeons in this study were all experienced at examining very young children. IOP readings were a priority as the primary purpose of the study was to evaluate safety. The investigators were each experienced in the measurement of IOP postoperatively in young children. Cell and flare were evaluated as precisely as possible given the age of the patients. A tabletop slit lamp was used when possible to make this assessment. In addition, a global inflammation assessment was added as this evaluation was deemed appropriate by the investigators for this age group. It included an assessment of conjunctival injection, cillary/limbal injection, chemosis, and hypopyon. It is acknowledged that although serious inflammatory complications would not have been overlooked, precise comparisons of the cell and flare response to surgery between groups may have been limited in this study of children aged 0–3 years. As a result of this, the efficacy comparison between the drugs studied is less precise than it would be in an adult study.

In conclusion, this study has demonstrated that the safety profile of topical difluprednate 0.05% is similar to that of topical prednisolone acetate 1%, both dosed four times daily, in the postoperative management of children up to 3 years of age who have undergone cataract surgery. This study supports the safety of difluprednate 0.05% for the management of inflammation in young children after cataract surgery.


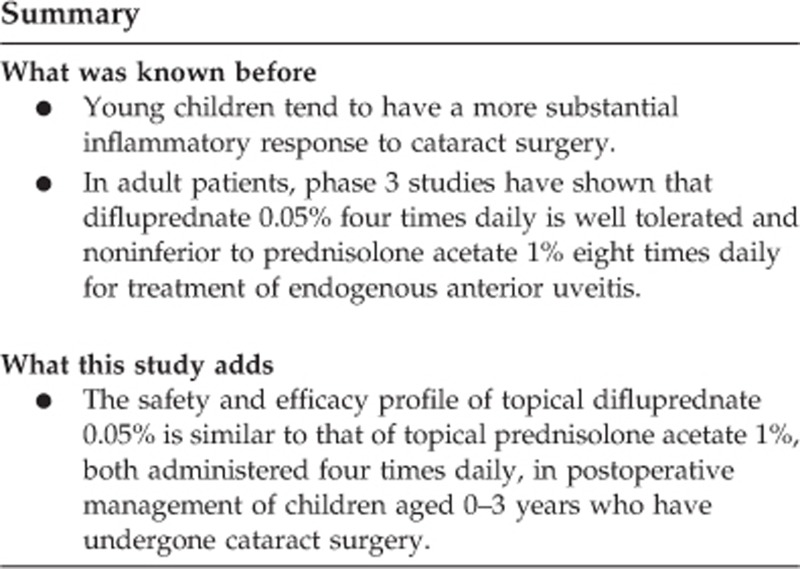


## Figures and Tables

**Table 1:** Patient characteristics (safety population)

*Characteristic,* n *(%)*	*Difluprednate 0.05% (*n=*39)*	*Prednisolone acetate 1% (*n=*40)*
*Age*		
0–27 Days	3 (7.7)	3 (7.5)
28 Days–23 months	28 (71.8)	26 (65.0)
24 Months–47 months^a^	8 (20.5)	11 (27.5)
		
*Sex*		
Male	17 (43.6)	20 (50.0)
Female	22 (56.4)	20 (50.0)
		
*Ethnicity*		
Hispanic, Latino, or Spanish	9 (23.1)	8 (20.0)
Other	30 (76.9)	32 (80.0)
		
*Race*		
White	21 (53.8)	24 (60.0)
Black or African American	9 (23.1)	9 (22.5)
Asian	0 (0.0)	1 (2.5)
Multi-racial	3 (7.7)	2 (5.0)
Other	6 (15.4)	4 (10.0)
		
*Iris colour*		
Brown	22 (56.4)	22 (55.0)
Green	0 (0.0)	1 (2.5)
Blue	15 (38.5)	16 (40.0)
Grey	1 (2.6)	0 (0.0)
Other	1 (2.6)	1 (2.5)

a The 24–47-month category included all patients who had reached their second birthdays even if they had reached their third birthday. Hence, some patients reached 47 months.

**Table 2:** Summary of adverse events (safety population)

*AE category,* n *(%)*	*Difluprednate 0.05% (*n=*39)*	*Prednisolone acetate 1% (*n=*40)*
Death	0 (0.0)	0 (0.0)
Serious AE	8 (20.5)	11 (27.5)
Discontinuation due to AE	0 (0.0)	0 (0.0)
*⩾1 AE (related or not related to study medication)*	29 (74.4)	30 (75.0)
Conjunctivitis	3 (7.7)	0 (0.0)
Posterior capsule opacification	3 (7.7)	0 (0.0)
Eye inflammation	0 (0.0)	2 (5.0)
Pyrexia	0 (0.0)	2 (5.0)
Nasopharyngitis	5 (12.8)	2 (5.0)
Ear infection	3 (7.7)	1 (2.5)
Sinusitis	2 (5.1)	0 (0.0)
Medical observation	6 (15.4)	10 (25.0)
Intraocular pressure increased	3 (7.7)	1 (2.5)
Hypotonia	0 (0.0)	2 (5.0)
Rash	1 (2.6)	2 (5.0)
Dermatitis diaper	0 (0.0)	2 (5.0)
Cataract operation (non-study eye)	3 (7.7)	6 (15.0)
⩾1 AE related to treatment (adverse drug reaction)	3 (7.7)	2 (5.0)
Corneal oedema	1 (2.6)	0 (0.0)
Ocular hypertension	0 (0.0)	1 (2.5)
Intraocular pressure increased	2 (5.1)	1 (2.5)

Abbreviation: AE, adverse event.

**Table 3:** Global assessment of inflammation by visit (intent-to-treat population)

*Visit*	*Assessment score,* n *(%)*	*Difluprednate 0.05% (*n=*39)*	*Prednisolone acetate 1% (*n=*40)*
Day 1	Total	39	40
	Clear	12 (30.8)	7 (17.5)
	Improving satisfactorily	27 (69.2)	33 (82.5)
	Not improving or worsening	0 (0.0)	0 (0.0)
Day 8	Total	39	40
	Clear	19 (48.7)	10 (25.0)
	Improving satisfactorily	19 (48.7)	28 (70.0)
	Not improving or worsening	1 (2.6)	2 (5.0)
Day 15	Total	39	40
	Clear	22 (56.4)	20 (50.0)
	Improving satisfactorily	17 (43.6)	20 (50.0)
	Not improving or worsening	0 (0.0)	0 (0.0)
Day 29	Total	39	40
	Clear	31 (79.5)	29 (72.5)
	Improving satisfactorily	8 (20.5)	10 (25.0)
	Not improving or worsening	0 (0.0)	1 (2.5)
1 Week after last dose	Total	39	40
	Clear	35 (89.7)	36 (90.0)
	Improving satisfactorily	3 (7.7)	3 (7.5)
	Not improving or worsening	1 (2.6)	1 (2.5)
3 Months after last dose	Total	39	40
	Clear	36 (92.3)	37 (92.5)
	Improving satisfactorily	2 (5.1)	3 (7.5)
	Not improving or worsening	1 (2.6)	0 (0.0)

**Table 4:** Individual components of the global assessment of inflammation at day 15 (intent-to-treat population)

*Individual components of global assessment of inflammation*	*Patients with grade 0,* n *(%)*	
	*Difluprednate 0.05% (*n=*39)*	*Prednisolone acetate 1% (*n=*40)*
Anterior chamber cell	30 (78.9)	31 (77.5)
Anterior chamber flare	29 (74.4)	28 (70.0)
Corneal clarity	37 (94.9)	36 (90.0)
Conjunctival injection	35 (89.7)	37 (92.5)
Ciliary/limbal injection	39 (100.0)	40 (100.0)
Chemosis	39 (100.0)	39 (97.5)
Hypopyon	39 (100.0)	40 (100.0)
Vitritis	39 (100.0)	39 (97.5)
Wound integrity	39 (100.0)	40 (100.0)
Photophobia	38 (97.4)	38 (95.0)
Lacrimation	39 (100.0)	38 (95.0)
