# Supplementary figures and images for: Difluprednate versus prednisolone acetate for inflammation following cataract surgery in pediatric patients: a randomized safety and efficacy study
Source: Eye (Lond). 2016 Jul 1;30(9):1187–94. doi: 10.1038/eye.2016.132 (PMC5023802; doi:10.1038/eye.2016.132)

**Supplementary Figure 1**

**
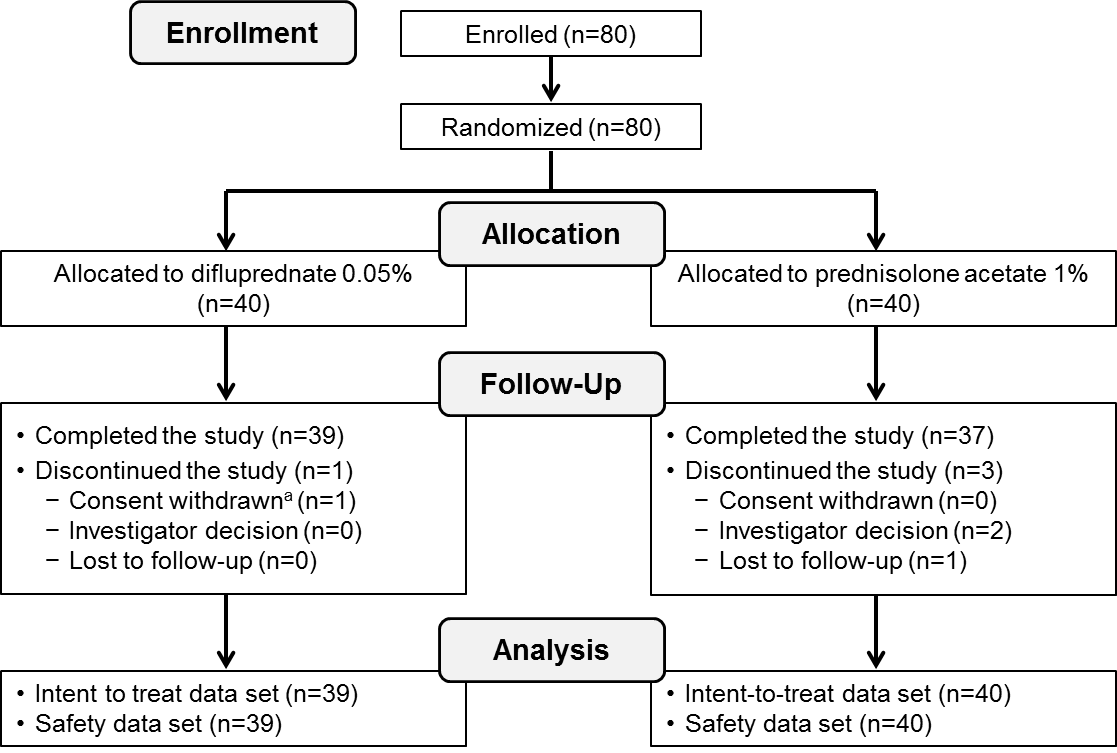
**

Supplement: Supplementary Information [file eye2016132x1.doc]
